# Pesticide exposure assessed through agricultural crop proximity and risk of amyotrophic lateral sclerosis

**DOI:** 10.1186/s12940-017-0297-2

**Published:** 2017-08-29

**Authors:** Marco Vinceti, Tommaso Filippini, Federica Violi, Kenneth J. Rothman, Sofia Costanzini, Carlotta Malagoli, Lauren A. Wise, Anna Odone, Carlo Signorelli, Laura Iacuzio, Elisa Arcolin, Jessica Mandrioli, Nicola Fini, Francesco Patti, Salvatore Lo Fermo, Vladimiro Pietrini, Sergio Teggi, Grazia Ghermandi, Renato Scillieri, Caterina Ledda, Cristina Mauceri, Salvatore Sciacca, Maria Fiore, Margherita Ferrante

**Affiliations:** 1 Environmental, Genetic, and Nutritional Epidemiology Research Center - CREAGEN, Department of Biomedical, Metabolic and Neural Sciences, University of Modena and Reggio Emilia, 287 Via Campi, 41125 Modena, Italy; 2 Department of Epidemiology, Boston University School of Public Health, 715 Albany Street, Boston, MA 02118 USA; 3 Research Triangle Institute, Research Triangle Park, 3040 E Cornwallis Road, Durham, NC 27709 USA; 4 Department of Engineering “Enzo Ferrari”, University of Modena and Reggio Emilia, 10 Via Vivarelli, 41125 Modena, Italy; 5 Department of Biomedical, Biotechnological, and Translational Sciences, University of Parma, 14 Via Gramsci, 43126 Parma, Italy; 6 School of Medicine, University Vita-Salute San Raffaele, 58 Via Olgettina Milano, 20132 Milan, Italy; 7 Department of Neuroscience, S. Agostino-Estense Hospital, and University of Modena and Reggio Emilia, 1355 Via Pietro Giardini, 41126 Modena, Italy; 8 Department of Medical, Surgical Sciences and Advanced Technologies “G.F. Ingrassia”, University of Catania, 87 Via S. Sofia, 95123 Catania, Italy; 9 Neurologic Unit, AOU Policlinico - Vittorio Emanuele, University of Catania, 628 Via Plebiscito, 95124 Catania, Italy; 10 Department of Neuroscience, Neurology Unit, University of Parma, 14 Via Gramsci, 43126 Parma, Italy; 11 Department of Prevention, Catania Local Health Unit, 5 Via Santa Maria la Grande, 95124 Catania, Italy

**Keywords:** Amyotrophic lateral sclerosis, Pesticides, Case-control study, Epidemiology, Risk

## Abstract

**Background:**

Epidemiologic studies have raised the possibility that some pesticide compounds induce the neurodegenerative disease amyotrophic lateral sclerosis (ALS), though the available evidence is not entirely consistent.

**Methods:**

We conducted a population-based case-control study in two Italian populations to assess the extent to which residence in the vicinity of agricultural crops associated with the application of neurotoxic pesticides is a risk factor for ALS, using crop acreage in proximity to the residence as an index of exposure.

**Results:**

Based on 703 cases and 2737 controls, we computed an ALS odds ratio of 0.92 (95% confidence interval 0.78-1.09) for those in proximity to agricultural land. Results were not substantially different when using alternative exposure categories or when analyzing specific crop types, with the exception of a higher risk related to exposure to citrus orchards and olive groves in Southern Italy, though based on few exposed subjects (*N* = 89 and 8, respectively). There was little evidence of any dose-response relation between crop proximity and ALS risk, and using long-term residence instead of current residence did not substantially change our estimates.

**Conclusions:**

Though our index of exposure is indirect and subject to considerable misclassification, our results offer little support for the hypothesis that neurotoxic pesticide exposure increases ALS risk.

## Background

Amyotrophic Lateral Sclerosis (ALS) is a fatal neurodegenerative disease characterized by a rapidly progressive degeneration of the upper and lower motor neurons [1, 2]. ALS is traditionally classified into familial and sporadic forms, the latter being much more common. Its incidence in Europe is two to three cases per 100,000 person-years, with heterogeneous distribution worldwide [1, 3].

The etiology of ALS is largely unknown. As with any disease, there is presumed to be an interaction between genes and environmental factors [4]. Although recent genetic studies have improved our understanding of both familial and sporadic forms of the disease, the contribution of environmental factors has been much more difficult to assess [2, 5–9]. Among the proposed environmental risk factors, there are hypotheses relating to neurotoxic chemicals such as heavy metals, the metalloid selenium, and some pesticide categories [6, 10–12]. Pesticides, in particular, have been extensively investigated in epidemiologic studies [13, 14]. Some of these studies reported evidence of an association, but results were inconsistent and subject to possible selection bias, differential recall, confounding and exposure misclassification [15–17]. Most studies have focused on occupational pesticide exposure; thus, little is known about the role of non-occupational exposure, which may occur through ingestion, dermal contact and inhalation. Non-occupational exposure may come from drift while applying pesticides to agricultural fields as well as from their volatilization after application [18–25], depending on the specific pesticides used and the meteorological conditions [26–28]. Residential ‘passive’ exposure to pesticides occurring proximal to agricultural areas, measured by estimating crop acreage around a residence through remote sensing, land cover maps and geographical information systems [21, 29, 30], has received considerable attention in epidemiologic research in relation to various diseases [31–35]. In the present population-based case-control study, we assessed the extent to which residential proximity to agricultural land in general, and to specific crop types, could be a risk factors for ALS. The study was conducted in two Italian regions, one from the North and the other from the South of the country.

## Methods

### Study population

Following approval of the Ethics Committees of the Modena and the Catania provinces, we designed a population-based case-control study to investigate the role of environmental risk factors in ALS etiology [36]. We attempted to identify all cases of ALS newly-diagnosed during 1998-2011 among residents in three provinces, Parma, Reggio Emilia and Modena, of the Emilia-Romagna region in Northern Italy, and in Catania, a province in Sicily. These provinces encompass an overall population of around 3,000,000 and have, as far as the three Emilia-Romagna provinces are concerned, very similar orography, land use patterns and percentage of population employed in agriculture [37]. In fact, at the last Agricultural General Census carried out in 2010, percentage of agricultural land use was 42.2, 44.1 and 44.6 in Parma, Reggio Emilia and Modena, respectively, while the corresponding percentage of employees in agriculture was 9.0, 9.7 and 11.6 [37]. We used several available administrative data sources to identify patients diagnosed in these provinces, known to have rather similar ALS incidence rates [38, 39]: the ALS Emilia-Romagna Registry (established in 2009), the hospital discharge records for the entire study period released by the Emilia-Romagna and Sicily regions, the drug prescription directories and the death certificate files. Only ‘definite’ and ‘probable’ ALS cases, as defined by using El Escorial revised criteria [40], were included in the study. For each enrolled patient, we randomly selected four controls from the database of National Health Service directory, to which all Italian residents are included. Controls were individually matched to patients by sex, year of birth, and province of residence, and in the Reggio Emilia province, by calendar year. Matching by calendar year was not feasible in the other provinces.

### Exposure assessment

To assess non-occupational pesticide exposure, we used the proxy variable of land use and specifically crop density close to the subject’s home. This measure is premised on the evidence that direct pesticide drift and the aerial volatilization and spreading of these compounds following their applications in fields leads to residential exposure. The aerial transport of a pollutant from its source to a point of exposition is usually assessed using atmospheric dispersion models [41, 42]. However, in the present study the complexity of the sources (hundreds of fields with pesticides emission rate not quantifiable) prevented the use of such models. We therefore assessed exposure using a methodology based on remote sensing, land use, and residential history within a geographical information system [29, 43], as recently described in detail on a study on pesticide exposure and childhood leukemia risk in the proximity of agricultural applications [34]. We ascertained the residential address of cases at the date of diagnosis and of matched controls as well as their oldest ascertainable residence for which exposure data were also available (‘historical residence’), provided that such residence was subsequently stable for at least two years (otherwise the subject was excluded from the analysis). We used the Ministry of Finance Revenue Agency database to abstract this historical residence, which was as of December 1979 for the Modena, Reggio Emilia and Parma residents, and as of December 1989 for Catania (no information about land use in Sicily was available before that year). We also abstracted, from the Ministry of Finance database, information about any agricultural business activity since 1979 engaged in by the participants in Modena and Reggio Emilia. We geocoded participants’ addresses using the database available at the Province Authorities of Modena, Reggio Emilia and Parma, which had been already validated in previous studies [44, 45]. For addresses that could not be retrieved in these databases (around 10%), we used Google Earth to geocode, and for the few participants for whom the address could still not be retrieved (*N* = 120), we directly geocoded it in loco using a portable GPS device (Garmin GPSmap 60CSx, Garmin Int. Corp., Olathe, KS). We included all geocoded addresses in a Geographical Information System, using ARC-GIS software (version 10, ESRI, Redlands, CA 2010).

We also obtained data on land use from aerial photos made available by the Department of Agriculture of the Emilia-Romagna and Sicily Regions. For the land within a circular 100-m zone around each participant’s residence, we determined the proportion classified as urban, rural, or wetlands, as well as the specific crop types according to a detailed classification [46]. Since aerial photos of the regions were taken and the related land cover map made available only about once in each decade or even less, we used the 2003 photo and map for Emilia-Romagna and the 2009 photo and map for Sicily to assess ‘recent exposure,’ i.e. that linked to the 1998-2011 period of disease diagnosis of the ALS cases, and the oldest ones available to assess long-term antecedent (‘historical’) exposure, i.e. that for 1976 for Emilia-Romagna and for 1989 for Sicily. Using these land use maps, we determined the crop density for overall agricultural land and for the categories of vineyards, orchards, arable crops (cereals grown by arable farming) and vegetable crops. For Catania, we also added olive groves among the crops investigated. There were some inconsistencies in the land use classification: the crop types ‘arable’ and ‘vegetable’ were not split in the historical exposure for Sicily, and no information about citrus orchards (a Sicily-specific crop type) was available for the recent period. Finally, and consistent with other studies in this field, we also recalculated exposures using a zone of 1000 m around the subject’s residence, instead of 100 m, and an Inverse Distance Weighting (IDW) method:

$$ E=\frac{\sum_i{w}_i{a}_i}{\sum_i{w}_i} $$

where *E* is the exposure, *a*_*i*_ is the area of the *i-th* field inside the exposition circle (1000 m) and *w*_*i*_ a weighting factor based on the inverse of the subject-field distance.

In the Geographical Information System, we also considered the geocoded data of the pesticide-free farms of the Emilia-Romagna and Sicily regions [47], removing from the area considered as ‘agricultural’ the corresponding area.

We collected information about the specific pesticides or pesticide categories that were generally used for each crop type. To do that, we obtained a list of all compounds used for the different crop types of the regional territory from the Department of Agriculture of the Emilia-Romagna and Sicily Regions. We then validated the real use of these pesticide categories for the most common crop types with the help of technicians of Emilia-Romagna Department of Agriculture, who assessed actual pesticide use in a sample of 118 farms in 2003, by scrutinizing the ‘Registers of treatment,’ completed according to the Italian legislation from each farm. This register included details of type, timing, and amount of pesticide application. Finally, we assessed the literature for data on the neurotoxicity of the compounds used for the crop types identified in the study area [48–53]. In Table 1, we report the results of such a search, listing the pesticide categories associated with each crop type and which were characterized by neurotoxicity effects according to literature data [48–53].

**Table 1:** Pesticides with neurotoxic properties used for the crops identified from land use map in the study area (I = insecticides; H = herbicides; F = fungicides)

Crops	Pesticides
Vineyards	organophosphates (I/H); triazoles (F); phenilamides (F)
Orchards	organophosphates (I/H); triazoles (F); phenilamides (F); neonicotinoids (I)
Citrus orchards	organophosphates (I/H); neonicotinoids (I);
Arable crops	2,4D (H); MCPA (H); dicamba (H); azatrine (H); pyrethroids (I); glyphosate (H)
Vegetables crops	neonicotinoids (I); pyrethroids (I); phenilamides (F)
Olive groves	organophosphates (I/H); neonicotinoids (I); glyphosate (H)

### Data analysis

We estimated the crude and pooled odds ratio for ALS associated with exposure to pesticides, using Episheet [54]. We also used conditional logistic regression using dichotomous exposure categorization (>0 crop type in the vicinity vs. 0), continuous data (for 20% increase in crop percentage), tertiles of exposure and three a priori defined crop percentage-based categories: <10%; 10%-30%; and >30%. Given that many subjects had no rural land use within the 100 m zone around their residence, tertiles were computed temporarily excluding those with zero exposure and then adding those with zero exposure back into the lowest category. We also carried out stratified analyses by age group and by region, and using a zone of 1000 m and the IDW algorithm instead of 100 m around each subject’s home.

## Results

We identified 703 cases of ALS diagnosed during the study period 1998-2011, 499 in Emilia-Romagna and 204 in Sicily. We then identified 2814 matched controls, but we could not include all of them in the analysis, due to erroneous personal data or lack of effective residence in the province at the date of interest. Ultimately, 2737 matched controls (1935 in Emilia-Romagna and 802 in Sicily) were included. 537 cases and 2049 controls were found to be resident in the study area in 1976 for the Emilia-Romagna region and in 1989 for Sicily, and therefore could be retained in the historical analysis. 52% of study subjects were male, and most of them (97.8%) were born in Italy. The mean age at disease onset was 65.9 years, with values of 62.9 years and 67.2 years in Sicily and Emilia-Romagna region, respectively. The distribution of sex, age, province of residence, and country of birth of study participants is reported in Table 2.

**Table 2:** Characteristics of study participants. Number (%) are presented

	Cases	Controls	Total
Sex			
Male	365 (51.6)	1413 (51.9)	1778 (51.7)
Female	337 (48.4)	1324 (48.1)	1662 (48.3)
Age			
Mean (SD)	65.9 (12.0)	66.0 (11.9)	66.0 (11.9)
< 65 years	281 (40.0)	1088 (39.7)	1369 (39.8)
≥ 65 years	422 (60.0)	1649 (60.3)	2071 (60.2)
Region			
Emilia Romagna	499 (71.0)	1935 (70.7)	2434 (70.8)
Parma	112 (15.9)	431 (15.7)	543 (15.8)
Reggio Emilia	152 (21.6)	599 (21.9)	751 (21.8)
Modena	235 (33.4)	905 (33.1)	1140 (33.1)
Sicily (Catania)	204 (29.0)	802 (29.3)	1006 (29.2)
Birth country			
Italian	682 (97.0)	2681 (98.0)	3363 (97.8)
Foreign	21 (3.0)	56 (2.0)	77 (2.2)
Total	703	2737	3440

Overall, there were 290 cases (41.3%) and 1184 controls (43.3%) who had some agricultural crops in the vicinity (≤ 100 m) of their homes. Distribution of matched case-control sets by recent and historical exposure to total agricultural land is reported in Table 3. There was little association between ALS risk and proximity to any agricultural land in the overall study area (OR 0.92, 95% CI 0.78-1.09) and within each region (Table 4). The same was true when specific crop types were considered, except for a statistically unstable increased risk linked to vegetable crops in both study regions, which was based on only 8 exposed subjects, and an association seen just for Sicily with respect to vineyards, orchards and olive groves. The latter effect estimates, however, were statistically unstable except for orchards, to which 121 subjects (37 cases and 84 controls) were exposed, while the corresponding figures for vineyards, vegetable crops and olive groves were 5, 2 and 17, respectively. In continuous analysis, the OR associated with 20% increase in acreage was close to unity in the overall study area for total agricultural land and for most crop types, except for the imprecise estimates for proximity to vegetable crops (OR 4.00, 95% CI 0.25-63.95). In area-specific analysis, an increase of 20% of acreage was strongly associated with excess ALS risk only for vegetable crops exposure in the Emilia-Romagna region, and for total agricultural land, orchards and particularly olive groves (OR 5.01, 95% CI 1.05-23.97) in Sicily.

**Table 3:** Distribution of individually matched case-control sets according to the number of matched controls and the exposure of the case and the matched controls to agricultural land

Matching ratio		Exposed controls				
		0	1	2	3	4
1:2	Case exposed	1	1	0	-	-
	Case unexposed	1	1	0	-	-
1:3	Case exposed	3	15	12	0	-
	Case unexposed	8	16	11	2	-
1:4	Case exposed	21	82	89	59	7
	Case unexposed	42	126	128	60	18

**Table 4:** Odds ratios (OR) of amyotrophic lateral sclerosis associated with current proximity to any agricultural land or specific crop type within 100 m from residence (A), and with 20% continuous increase of any agricultural land or specific crop type (B)^a^

	Cases	Controls	Total (*N* = 3440)		Cases	Controls	Emilia-Romagna (*N* = 2434)		Cases	Controls	Sicily (*N* = 1006)	
Crops	Exp/Unexp	Exp/Unexp	OR	(95% CI)	Exp/Unexp	Exp/Unexp	OR	(95% CI)	Exp/Unexp	Exp/Unexp	OR	(95% CI)
(A)												
Total agricultural land	290/413	1184/1553	0.92	(0.78, 1.09)	205/294	858/1077	0.88	(0.72, 1.07)	85/119	326/476	1.04	(0.76, 1.42)
Vineyards	27/676	91/2646	1.16	(0.75, 1.80)	25/474	88/1847	1.11	(0.70, 1.75)	2/202	3/799	2.64	(0.44, 15.89)
Orchards	46/657	181/2556	0.99	(0.71, 1.38)	9/490	97/1838	0.35	(0.17, 0.69)	37/167	84/718	1.89	(1.24, 2.89)
Arable crops	203/500	826/1911	0.94	(0.78, 1.13)	191/308	756/1179	0.97	(0.79, 1.18)	12/192	70/732	0.65	(0.35, 1.23)
Vegetable crops	3/700	5/2732	2.35	(0.56, 9.82)	2/497	4/1931	1.94	(0.35, 10.64)	1/203	1/801	3.95	(0.25, 63.36)
Olive groves									6/198	11/791	2.18	(0.80, 5.96)
(B)												
Total agricultural land			1.01	(0.94, 1.09)			0.96	(0.88, 1.04)			1.35	(1.13, 1.63)
Vineyards			1.15	(0.86, 1.54)			1.12	(0.83, 1.51)			Too high	
Orchards			0.93	(0.70, 1.24)			0.12	(0.02, 0.82)			1.52	(1.08, 2.13)
Arable crops			0.98	(0.89, 1.08)			0.98	(0.89, 1.08)			1.01	(0.63, 1.61)
Vegetable crops			4.00	(0.25, 63.9)			4.00	(0.25, 63.95)			-	
Olive groves											5.01	(1.05, 23.97)

^a^Results for dichotomous analysis expressed as crude OR for matched sets, and for continuous analysis using a conditional logistic regression model. Some estimates could not be computed because of too few exposed subjects

When we categorized exposure into tertiles (Table 5), in the overall population, recent exposure to vineyards was associated with an increased risk in the intermediate category, but with a decreased risk in the highest category. No other crop type in this analysis was associated with any meaningful trend in risk, including vegetable crops. A similar picture was observed when limiting the analysis to the Emilia-Romagna region, while for Sicily a higher and statistically unstable effect estimate for the top exposure category was observed for total agricultural land, vineyards, orchards, and olive groves, although only orchards showed a dose-response relation. Results did not differ much within age or sex categories, or when the 100 m zone was replaced with the 1000 m zone, or when the IDW method or a priori defined exposure categories were used (data not shown).

**Table 5:** Odds ratio (OR) of amyotrophic lateral sclerosis for current proximity to agricultural fields in tertiles of current exposure^a^

	Total					Emilia-Romagna					Sicily				
	I	II		III		I	II		III		I	II		III	
Total agricultural land															
Cut off (%)	<12.2	12.2-38.8		>38.8		<17.2	17.2-44.6		>44.6		<6.3	6.3-21.6		>21.6	
Cases/Controls	487/1949	113/394		103/394		354/1363	80/286		65/286		138/586	25/108		41/108	
OR (95% CI)	1.00	1.15	(0.91, 1.45)	1.04	(0.82, 1.33)	1.00	1.08	(0.82, 1.43)	0.88	(0.65, 1.18)	1.00	0.97	(0.60, 1.57)	1.57	(1.06, 2.34)
Vineyards															
Cut off (%)	<9.7	9.7-31.1		>31.1		<9.8	9.8-31.5		>31.5		<2.8	2.8-9.4		>9.4	
Cases/Controls	683/2677	11/30		9/30		480/1877	11/29		8/29		203/800	0/1		1/1	
OR (95% CI)	1.00	1.46	(0.72, 2.95)	1.19	(0.55, 2.54)	1.00	1.51	(0.74, 3.07)	1.10	(0.49, 2.44)	1.00	-		3.47	(0.22, 55.83)
Orchards															
Cut off (%)	<7.8	7.8-26.4		>26.4		<9.6	9.6-28.9		>28.9		<7.5	7.5-23.4		>23.4	
Cases/Controls	675/2618	15/59		13/60		497/1871	1/33		1/31		179/746	12/29		13/27	
OR (95% CI)	1.00	0.98	(0.55, 1.74)	0.85	(0.46, 1.57)	1.00	0.11	(0.02, 0.82)	0.11	(0.02, 0.84)	1.00	1.74	(0.86, 3.49)	1.94	(1.00, 3.77)
Arable crops															
Cut off (%)	<13.1	13.1-38.7		>38.7		<14.9	14.9-40.9		>40.9		<3.9	3.9-17.1		>17.1	
Cases/Controls	551/2187	89/275		63/275		359/1431	82/252		58/252		196/756	2/23		6/23	
OR (95% CI)	1.00	1.29	(0.99, 1.69)	0.91	(0.68, 1.22)	1.00	1.31	(0.99, 1.73)	0.91	(0.67, 1.24)	1.00	0.35	(0.08, 1.48)	1.01	(0.40, 2.54)
Vegetable crops															
Cut off (%)	<6.4	6.4-20.9		>20.9		<8.1	8.1-20.9		>20.9		<4.8	-		>4.8	
Cases/Controls	701/2734	1/2		1/1		498/1933	0/1		1/1		203/801	0/0		1/1	
OR (95% CI)	1.00	1.81	(0.16, 20.08)	4.00	(0.25, 63.95)	1.00	-		4.00	(0.25, 63.95)	1.00	-		3.46	(0.22, 55.78)
Olive groves															
Cut off (%)											<2.9	2.9-9.1		>9.1	
Cases/Controls											199/795	0/4		5/3	
OR (95% CI)											1.00	-		6.44	(1.54, 26.98)

^a^Tertile cutpoints based on exposure of subjects having proximity to crops >0, with subjects having exposure = 0 included in the bottom tertile. OR computed using a conditional logistic regression model. Some estimates could not be computed because of too few exposed subjects

Results of corresponding analyses for historical exposure are reported in Tables 6 and 7. Exposure to agricultural land considered as a dichotomous variable had a near null relation (OR 1.05, 95% CI 0.83-1.32), also seen in the area-specific analysis for Sicily but not for Emilia-Romagna (Table 6). Exposure to orchards was moderately associated with an excess risk in the overall study population, entirely due to citrus orchards in Sicily (OR 1.40, 95% CI 0.84-2.32 in dichotomous analysis). Exposure to vegetables crops in Emilia-Romagna region was associated with a statistically unstable excess risk, based on 5 exposed subjects only, and this was also true for exposure to olive groves in Sicily, based on 8 exposed subjects. Results were substantially confirmed in the analysis based on 20% continuous increase in exposure. In tertile-specific analyses (Table 7), there was substantially no evidence of dose-response relations both in the entire study population and in area-specific analyses, and the excess risk found in Sicily for citrus orchards and for olive groves was entirely confined to the highest tertile of exposure, based on 33 and 4 exposed subjects, respectively. These results did not change substantially when we limited the analyses to subjects who had been residing at the same address since 1979 (for Emilia-Romagna) or 1989 (for Sicily) until the date of diagnosis (for cases) or the corresponding calendar year (for matched controls).

**Table 6:** Odds ratios (OR) of amyotrophic lateral sclerosis associated with historical proximity to any agricultural land or specific crop type within 100 m from residence (A), and with 20% increase of crop type (B)^a^

	Cases	Controls	Total (*N* = 2235)		Cases	Controls	Emilia-Romagna (*N* = 1363)		Cases	Controls	Sicily (*N* = 872)	
Crops	Exp/Unexp	Exp/Unexp	OR	(95% CI)	Exp/Unexp	Exp/Unexp	OR	(95% CI)	Exp/Unexp	Exp/Unexp	OR	(95% CI)
(A)												
Total agricultural land	258/271	772/934	1.05	(0.83, 1.32)	224/119	661/359	1.02	(0.77, 1.33)	34/152	111/575	1.15	(0.75, 1.77)
Vineyards	28/501	122/1584	0.64	(0.41, 0.99)	28/315	121/899	0.64	(0.41, 0.99)	0/186	1/685	-	
All Orchards	40/489	124/1582	1.06	(0.72, 1.55)	16/327	55/965	0.82	(0.46, 1.47)	24/162	69/617	1.31	(0.79, 2.18)
Orchards (not citrus)	16/513	59/1647	0.77	(0.43, 1.38)	16/327	55/965	0.82	(0.46, 1.47)	0/186	4/682	-	
Citrus orchards									24/162	65/621	1.40	(0.84, 2.32)
Arable and Veg. crops	213/316	627/1079	1.04	(0.80, 1.35)	213/130	613/407	1.11	(0.85, 1.44)	0/186	14/672	-	
Arable crops					211/132	611/409	1.08	(0.83, 1.41)				
Vegetables crops					2/341	3/1017	2.67	(0.45, 15.96)				
Olive groves									3/183	5/681	2.23	(0.53, 9.35)
(B)												
Total agricultural land			1.05	(0.98, 1.12)			1.02	(0.94, 1.10)			1.18	(1.02, 1.36)
Vineyards			0.85	(0.60, 1.20)			0.86	(0.60, 1.23)			-	
All Orchards			1.13	(0.96, 1.34)			0.91	(0.60, 1.37)			1.20	(1.00, 1.44)
Orchards (not citrus)							0.91	(0.60, 1.37)			-	
Citrus orchards											1.23	(1.02, 1.48)
Arable and Veg. crops			1.03	(0.95, 1.12)			1.04	(0.95, 1.13)			-	
Arable crops							1.03	(0.94, 1.12)				
Vegetables crops							-					
Olive groves											Too high	

^a^Results for dichotomous analysis expressed as crude OR for matched sets, and for continuous analysis using a conditional logistic regression model. Some estimates could not be computed because of too few exposed subjects

**Table 7:** Odds ratios (OR) of amyotrophic lateral sclerosis for historical proximity to agricultural fields in tertiles of current exposure^a^

	Total					Emilia-Romagna					Sicliy				
	I	II		III		I	II		III		I	II		III	
Total agricultural land															
Cut off (%)	<28.1	28.1-77.8		>77.8		<30.8	30.8-78.4		78.4		<11.2	11.2-74.7		>74.7	
Cases/Controls	339/1192	98/257		92/257		190/580	78/220		75/220		156/612	15/37		15/37	
OR (95% CI)	1.00	1.27	(0.96, 1.69)	1.20	(0.91, 1.60)	1.00	1.09	(0.80, 1.50)	1.04	(0.76, 1.42)	1.00	1.51	(0.81, 2.82)	1.60	(0.86, 2.99)
Vineyards															
Cut off (%)	<12.4	12.4-25.1		>25.1		<12.4	12.4-24.0		>24.0		<0.1	0.1-99.9		>99.9	
Cases/Controls	507/1625	12/41		10/40		321/940	11/40		11/40		186/685	0/1		0/0	
OR (95% CI)	1.00	0.79	(0.40, 1.55)	0.77	(0.38, 1.56)	1.00	0.76	(0.38, 1.54)	0.81	(0.41, 1.61)	-				
All Orchards															
Cut off (%)	<8.2	8.2-41.0		>41.0		<5.4	5.4-32.5		>32.5		<13.7	13.7-63.5		>63.5	
Cases/Controls	499/1624	14/41		16/41		332/985	8/17		3/18		168/640	6/23		12/23	
OR (95% CI)	1.00	1.00	(0.54, 1.88)	1.41	(0.78, 2.53)	1.00	1.16	(0.49, 2.74)	0.50		1.00	0.91	(0.36, 2.30)	1.99	(0.97, 4.07)
Orchards (not citrus)															
Cut off (%)	>6.8	6.8-36.6		>36.6		<6.8	6.8-34.8		>34.8		<13.7	13.7-99.9		>99.9	
Cases/Controls	518/1667	8/20		3/19		332/985	8/17		3/18		186/684	0/2		0/0	
OR (95% CI)	1.00	1.01	(0.44, 2.34)	0.49	(0.14, 1.67)	1.00	1.16	(0.49, 2.74)	0.50	(0.15, 1.74)	-				
Citrus orchards															
Cut off (%)											<14.6	14.6-63.5		>63.5	
Cases/Controls											168/643	6/22		12/21	
OR (95% CI)											1.00	0.94	(0.37, 2.39)	2.19	(1.05, 4.54)
Arable and Veg. crops															
Cut off (%)	<25.7	25.7-65.7		>65.7		<27.3	27.3-65.9		>65.9		<7.1	7.1-24.6		>24.6	
Cases/Controls	381/1288	67/210		81/208		196/613	66/203		81/204		186/677	0/5		0/4	
OR (95% CI)	1.00	0.99	(0.72, 1.37)	1.21	(0.88, 1.66)	1.00	1.03	(0.74, 1.42)	1.26	(0.92, 1.76)	-				
Arable crops															
Cut off (%)						<27.3	27.3-65.9		>65.9						
Cases/Controls						196/614	66/203		81/203						
OR (95% CI)						1.00	1.03	(0.74, 1.42)	1.27	(0.92, 1.74)					
Vegetables crops															
Cut off (%)						<11.2	11.2-23.3		>23.3						
Cases/Controls						342/1018	1/1		0/1						
OR (95% CI)						1.00	4.01	(0.25, 64.11)	-						
Olive groves															
Cut off (%)											<8.5	8.5-18.6		>18.6	
Cases/Controls											183/683	0/2		3/1	
OR (95% CI)											1.00	*-*		12.01	(1.25, 115.45)

^a^Tertile cutpoints based on exposure of subjects having proximity to crops >0, with subjects having exposure = 0 included in the bottom tertile. OR computed using a conditional logistic regression model. Some estimates could not be computed because of too few exposed subjects

We also repeated all the above-mentioned analyses after restricting the study population to males and then to females, and to Reggio Emilia province, which was the only one for which we could sample controls from resident databases that were calendar-year-specific, allowing matching to cases. No evidence of substantial changes in effect estimates compared with the overall analysis emerged (data not shown).

## Discussion

Our study was designed to evaluate the hypothesis that residence near agricultural land, with presumed increased exposure to pesticides, is associated with an increased risk of ALS. Evidence of an association between pesticide exposure and ALS risk comes from human studies, including case reports [55–57] as well as epidemiologic studies, mainly of occupational exposure [1, 6, 7, 15–17, 58, 59]. In three epidemiologic investigations carried out in one of the regions included in the present study, Emilia-Romagna, we found an excess risk associated with pesticide exposure as assessed though a simple indicator of rural residence [60] or occupational pesticide exposure [61], but not when pesticide content in cerebrospinal fluid was used as biomarker of exposure [62]. Moreover, laboratory evidence on the relation between pesticides and ALS offers some biological plausibility for an association, through mechanisms such as altered protein synthesis, oxidative stress, inflammation, and mitochondrial dysfunction [17, 53, 63, 64].

Both environmental and biomonitoring studies indicate that residential proximity to agricultural fields increased exposure to various pesticide categories [18, 22, 65–67]. Residential proximity to specific crops might be even more relevant than usual diet and other sources in increasing the body burden of these contaminants [23], though the evidence is equivocal [19, 68].

Overall, we found little evidence that residence near agricultural land characterized by crop types for which pesticides, some of which with established neurotoxic properties, are used was associated with higher risk of ALS. This finding is consistent with there being little or no effect on ALS risk of pesticide and pesticide mixture drift in close proximity of the crops to which they are applied. Results were similar when we used either continuous indicators of crop use, tertiles or pre-set categories. In addition, it made little difference when we replaced the indicator of recent exposure, i.e. the crop acreage near home at time of diagnosis, with an indicator of historical exposure, under the hypothesis that passive exposure to neurotoxic pesticides and ALS development needs long induction and latent periods. The latter analysis also addresses exposure to pesticides that are no longer in use. Furthermore, results did not change substantially in the sensitivity analyses restricted to males or females (the latter being more likely to spend time at home), to residentially stable subjects, and to residents of the only province for which we could choose controls from calendar-year specific resident databases.

A partial exception to the overall null findings are some statistically imprecise excess risks associated with two crops in Sicily, citrus orchards and olive groves, though driven by a small number of exposed cases particularly for the latter crop type. These estimates of excess risk were the drivers of the estimate of excess risk associated with historical exposure to total agricultural land, which was almost entirely due to the increased risk in Sicily. Specific data for citrus orchards, a crop found only in Sicily in the present study, were available only for historical exposure, though the indication of an excess risk for overall orchards in the most recent period in the same population was most likely associated with such crop, given the large prevalence of this type of cultivation in Southern Italy. Regarding the excess risks associated with other crops, proximity to vineyards was not characterized by dose-response effects and was essentially null for historical exposure, while the excess risk associated with vegetable crops was based on a few subjects, only 2 cases and 3 controls. All of these crops were characterized by the use of neurotoxic pesticides such as organophosphates, triazoles and phenilamides for vineyards, pyrethroids, neonicotinoids and phenilamides for vegetables crops, and organophosphates and neonicotinoids for olive groves (in addition to glyphosate) and citrus orchards. However, some pesticides are commonly used for other crops, for which no increased risk was found, so these slight indications of an association are difficult to interpret. These excess risks were based on few exposed subjects and therefore had wide confidence intervals, except for orchards, for which there was limited evidence of a dose-response relation, particularly for long-term exposure.

Our largely null results might also indicate that the amount of exposure occurring ≤100 m from the points of pesticide application was too low to trigger disease onset and etiology. However, available data about pesticide environmental and biological monitoring in the proximity of agricultural fields indicate higher exposures of non-occupationally exposed subjects living in proximity, even at a larger distance than what we studied. Such sources of exposure, including dermal contact and consumption of locally-produced foods, may add to occupational exposure and broader dietary exposure. Factors such as time and length of application, meteorological conditions, time spent at home and in its immediate vicinity, and type of crops and applied pesticides, may have a major effect in modifying actual pesticide residential exposure in proximity to agricultural fields. In addition, although we could not collect specific occupational data for the study subjects, we expect that a much higher percentage of subjects living in the vicinity of agricultural land are occupationally involved in farming activity than those who live more distant from an agricultural field, and thus have a greater chance for occupational pesticide exposure [18, 69]. This expectation was confirmed by the data we collected for the subset of our study population that resided in Modena and Reggio Emilia provinces, which showed that percentage of ‘registered’ farmers was 11.1% and 2.5%, respectively, among the study subjects having and not having any agricultural land in the proximity of their residence. Consequently, the actual contrast in exposure between exposed and unexposed subjects in the present study was likely larger than what was estimated from our model. More accurate information about occupational exposure of study subjects was not available, due to the study design, which did not include the collection of direct information from participants, and due to the lack of availability of data from the National Social Insurance Agency.

To locate participants, we used the ‘official’ home address, which in some cases may differ from the actual address. Residential mobility in Italy, however, is much lower than some other countries [70]. In addition to exposure misclassification, the study may have been affected by unmeasured confounding. We were unable to control for socioeconomic or occupational status of our study subjects. The limited knowledge regarding the potential environmental, lifestyle, and genetic risk factors of ALS, particularly for the sporadic form, complicates the elimination of residual confounding. Conversely, there was evidence that the variables we used in the study for control sampling and data analyses were not important confounders. In fact, when we computed with the Episheet software a pooled OR of the matched sets conditioned on the matching factors the result was 0.92, and the crude result was also 0.92, indicating that collectively all the matching factors were uncorrelated with exposure and were therefore not confounders in this study.

Strengths of this study include inclusion of two different agricultural areas of Italy, characterized by different lifestyle and environmental characteristics, though with ALS rates comparable with those of other areas of Italy as well as US regions for which population-based data are available [38, 71–76]. The Northern Italy area we investigated experiences air pollution due to motorized traffic and other sources [77], while the area of Southern Italy included is generally characterized by a lower environmental pollution and by consumption of the well-known and healthy ‘Mediterranean diet’ [78]. Our results tended to be consistent across both areas. In addition, we used validated indicators of pesticide exposure, based on ad hoc surveys in a large number of farms in the study area, thus expecting a good reliability of the specific pesticide categories identified in the study areas. We were also able to take into account long-term antecedent pesticide exposure, even decades before disease onset, thus allowing for a long induction and latent period between exposure and ALS diagnosis. Finally, we avoided some selection bias, in that we did not have to obtain subjects’ consent to participate, as we used only information in the public domain.

We used a narrow zone of 100-m radius from each study subject’s home to define exposure status for study subjects according to the crop acreage near their residences. The narrow zone produced a more specific exposure classification than a wider zone would have done, at the price of some sensitivity [26, 79–82]. Larger exposure buffers in 250-1250 m range have been suggested and validated in other studies, taking into account pesticide drift and their volatilization from crops and soil after application [23, 25, 83–85]. Our choice was influenced by methods used to apply pesticides in the study area, which never include aerial sources such as in the US but only land-based devices. However, when we performed a supplementary analysis taking into account a larger buffer from the subjects’ residences, i.e. a 1000 m, results did not substantially change (data not shown).

## Conclusion

Overall, using a rough index of environmental exposure based on proximity of residence to agricultural land in two areas of Northern and Southern Italy, we found little association between this index and risk of ALS. Two specific crops, citrus orchards and olive groves, and their related pesticides, appear to deserve further investigation.

## Abbreviations

ALS: Amyotrophic lateral sclerosis
CI: Confidence interval
GPS: Global positioning system
IDW: Inverse distance weighting
OR: Odds ratio
